# Enhanced Defluoridation Using Novel Millisphere Nanocomposite of La-Doped Li-Al Layered Double Hydroxides Supported by Polymeric Anion Exchanger

**DOI:** 10.1038/s41598-018-29497-1

**Published:** 2018-08-06

**Authors:** Jianguo Cai, Yanyang Zhang, Yue Qian, Chao Shan, Bingcai Pan

**Affiliations:** 1 State Key Laboratory of Pollution Control and Resource Reuse, School of the Environment, Nanjing University, Nanjing, 210023 China; 2 Research Center for Environmental Nanotechnology (ReCENT), Nanjing University, Nanjing, 210023 China

## Abstract

A novel nanocomposite bead LaLiAl-LDH@201 was fabricated by doping a small amount of La into nanocrystalline Li/Al layered double hydroxides (LDHs) pre-confined inside polystyrene anion exchanger D201 (LiAl-LDH@201). A systematic characterisation of the resultant LaLiAl-LDH@201 (XRD, SEM-EDS, TEM-EDS, and XPS) evidenced the successful incorporation of La into the Li/Al LDHs, with their interlayer distance expanded to allow more exchangeable sites for fluoride uptake. The resultant LaLiAl-LDH@201 showed high and stable defluoridation performance over a wide range of pH from 4 to 9. The superior uptake capacity and affinity for fluoride of LaLiAl-LDH@201 over LiAl-LDH@201 were driven by both the increased anion exchange capacity of the embedded LDHs and the specific La-F interaction evidenced via XPS and TEM-EDS characterisation. Fixed-bed column test confirmed that the working capacity of LaLiAl-LDH@201 for defluoridation of authentic fluoride-rich groundwater was nearly twice that of LiAl-LDH@201. The fluoride-loaded LaLiAl-LDH@201 could be conveniently regenerated *in situ* by using NaOH + NaCl binary solution, achieving desorption efficiency above 98%. Moreover, negligible capacity loss, La leaching, or structure alteration was observed after five adsorption-regeneration cycles, indicating the high stability of LaLiAl-LDH@201. Therefore, the novel millisphere nanocomposite LaLiAl-LDH@201 was promising for efficient defluoridation from water and wastewater.

## Introduction

As generally known, excessive fluoride uptake from drinking water usually brings about health issues including skeleton fluorosis^1,2^. Severe fluoride contamination has been reported around the world. The concentration of fluoride in groundwater could reach as high as 8–30 mg/L in some locations in India and China^3–5^, much higher than the drinking water standard (1.5 mg/L) recommended by World Health Organisation (WHO).

Adsorptive removal of fluoride by activated alumina (AA) is one of the most widely used approaches to ensure the safety of drinking water^6–8^. However, many inherent defects of AA, such as narrow suitable pH range (4–5), chemical instability at acidic or basic pHs, ease of clogging during long-term operation, and difficulty in regeneration^7,9–11^, adversely affected the wider application of AA-based technology. In the past decade, Al-based layered double hydroxides (LDHs) have been developed as promising candidates for enhanced fluoride removal because the intercalated anions inside LDHs (usually Cl^−^) could be used for anion exchange with F^−^ ^12–15^. However, the powder nature of LDHs would induce problems during the solid/liquid separation process such as blockage and excessive pressure drop. Furthermore, the LDHs also suffer from metal leaching during the alkaline regeneration process. In our recent study^16^, a nanocomposite LiAl-LDH@201 with enhanced stability (over pH 3.5–12) and improved fluoride removal capacity was developed by impregnating Li/Al LDHs into a millimetric polystyrene anion exchanger. LiAl-LDH@201 inherited the mechanical strength and hydraulic properties from its polymeric host^17^ and could be regenerated for repeated use without significant capacity loss.

Recently, many studies suggested doping of high-valent metals such as La, Ce and Zr into metal oxides sorbents (such as AA, Fe_2_O_3_, etc.) could enhance their F removal capability^18–20^. Notably, rare earth elements (REEs) based adsorbents such as lanthanum based adsorbents (La(OH)_3_, La_2_O_3_) are widely reported to have high fluoride removal capacities, due to their high affinity for fluoride ion through ligand exchange^21–24^. For example, Dong and Wang^25^ developed a La-loaded magnetic cationic hydrogel composite with pronounced fluoride removal capacity at pH 2.8–4.0. Liu *et al*.^26^ fabricated a Al-humic acid-La aerogel composite exhibiting enhanced F removal capability even at circumneutral pH. Considering the relatively higher cost of REEs, many studies have been focusing on developing more cost-effective adsorbents via doping of REEs into inexpensive material such as metal oxides and chitosan^18,22,27,28^. Similarly, our very recent study^29^ demonstrated the superior defluoridation performance and stability of La-doped Li/Al LDHs over Li/Al LDHs to different extents at pH 5–9. Nevertheless, it is still imperative to address the limitations of such material, mainly the difficulties in solid/liquid separation and sustainable regeneration.

In this study, a novel millimetric nanocomposite La doped LiAl-LDH@201 (denoted as LaLiAl-LDH@201) was prepared to enhance the capacity and selectivity of the original sorbent. The resultant material was characterised with comprehensive techniques, and the defluoridation performance of LaLiAl-LDH@201 was evaluated with particular interest in the effects of pH and various competing anions. Furthermore, the promotional mechanism induced by La doping was elucidated. In addition, the performance of defluoridation from two practical groundwater samples (from Shandong and Yunnan Provinces) by LaLiAl-LDH@201 was evaluated via cyclic adsorption-regeneration in fixed-bed mode.

## Results and Discussion

### Characterisation of Adsorbents

**Figure 1:**
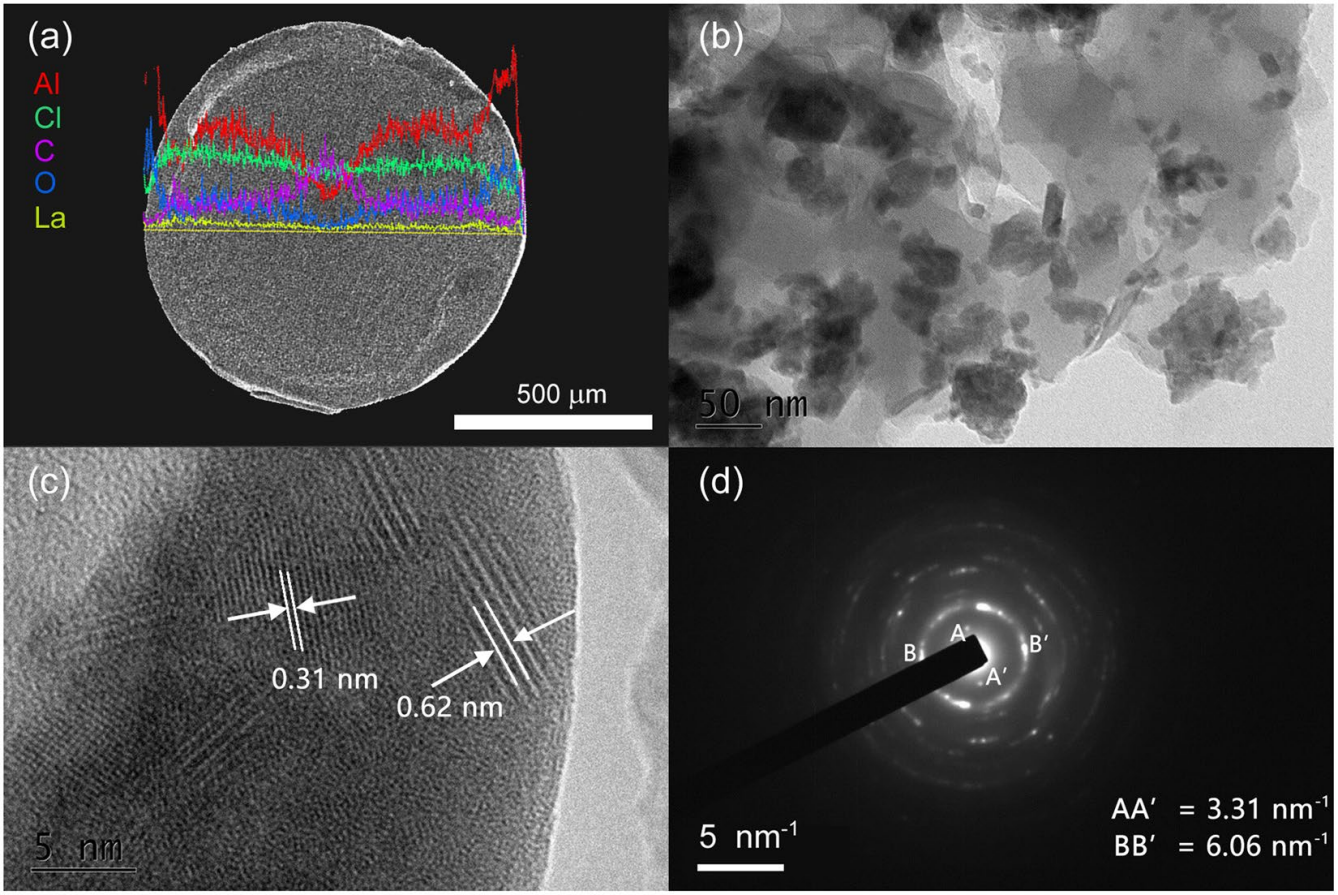
(**a**) Elemental distribution along the diameter on the cross section of the LaLiAl-LDH@201 bead on SEM-EDS. (**b**) TEM image of LaLiAl-LDH@201. (**c**) HR-TEM image of LaLiAl-LDH@201. (**d**) Selected area electron diffraction pattern of LaLiAl-LDH@201.

**Figure 2:**
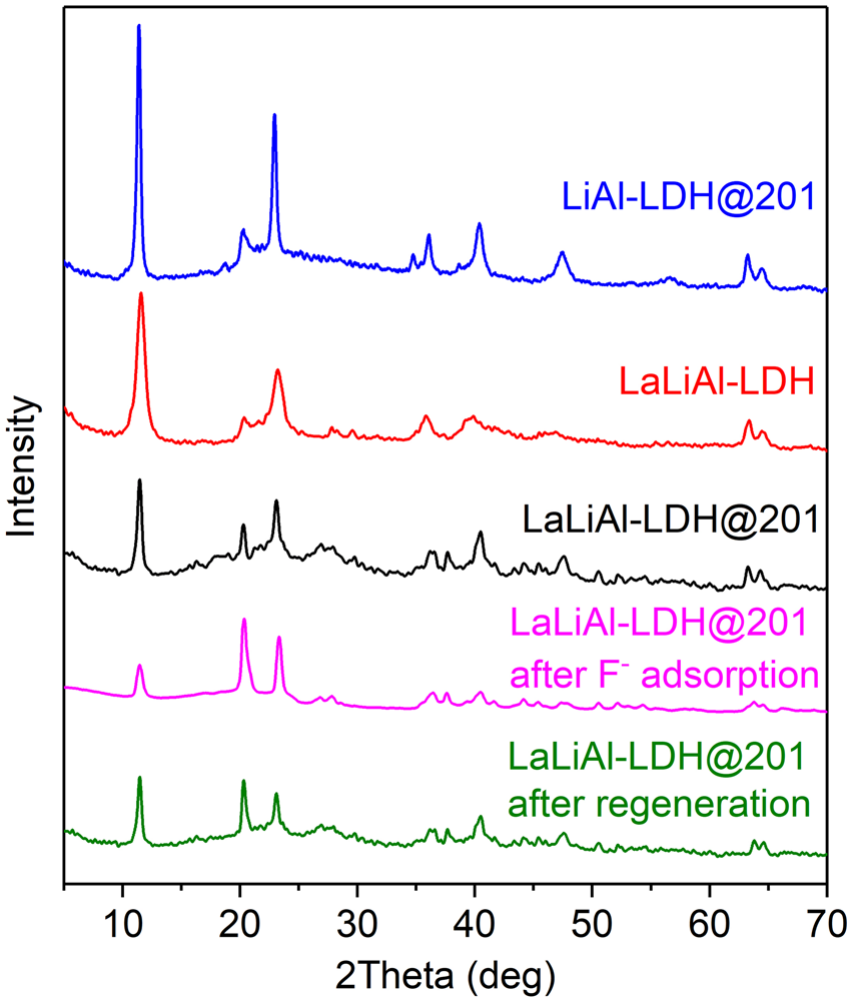
XRD patterns of LiAl-LDH@201, LaLiAl-LDH, and LaLiAl-LDH@201 (pristine, after F^−^ adsorption and after regeration).

The as-synthesised adsorbent LaLiAl-LDH@201 is present as spherical beads of 0.6–1.0 mm in diameter, with a large portion of La distributed in the outer sphere (Fig. 1a). The physio-chemical properties of LaLiAl-LDH@201 are summarised in Table S1. Compared with LiAl-LDH@201, insignificant changes between LiAl-LDH@201 and LaLiAl-LDH@201 in terms of pore volume, average pore diameter, and surface area were observed before and after La doping. The decrease in Al/Li molar ratio suggested the partial replacement of Al with La. The HR-TEM images of LaLiAl-LDH@201 are depicted in Fig. 1(b,c). The observed LDHs varied from spherical nanoparticles with clean edges to irregular state. More interestingly, two distinct electron diffraction patterns were observed with the inter-planar distance of 0.31 nm and 0.62 nm respectively (Fig. 1c), which matched its electron diffraction pattern with symmetric distances of 6.06 and 3.31 nm^−1^, respectively (Fig. 1d). The similar XRD patterns (Fig. 2) indicated that the LDH-based components in the three adsorbents were highly crystalline and the basic structure of LiAl-LDH was maintained during the doping with La. The diffraction peaks such as those at 11.5° (003), 23.1° (006), 20.3° (100) were assigned to the characteristic peaks of LiAl-LDH, agreeing with that of LiAl_2_(OH)_6_⋅*x*H_2_O (JCPDS card No. 31-0704). Comparatively, the peak of LiAl-LDH@201 at 34.8° was weakened in the pattern of LaLiAl-LDH@201, indicating certain distortion of the LDH structure due to the insertion of La. Besides, two extra peaks emerged at around 27.7° and 29.6° for LaLiAl-LDH@201 and were more pronounced for LaLiAl-LDH. Such extra peaks could be attributed to La(OH)_3_ (JPCDS card No. 36-1481), and were consistent with the observation of the 0.31 nm inter-planer distance in the lattice fringes. All the above analysis implied that some proportion of the doped La was present in the form of its (hydr)oxides, while the rest proportion was infused into the Li/Al-LDH via substitution for Al. Similar distribution of the dopant element was also reported for the insertion of lanthanide(III) cations into the ZnAl-LDHs^30^. As depicted in Fig. S1, the replacement of La^3+^ for Al^3+^ in the octahedron of metal layer would result in an expansion of the interlayer distance^30,31^ due to the significantly larger ionic radius of La^3+^ (116 pm) over Al^3+^ (39 pm)^32^. Our recent study also found that the pore diameter of LaLiAl-LDH was significantly larger than that of LiAl-LDH^29^ possibly due to La doping.

**Figure 3:**
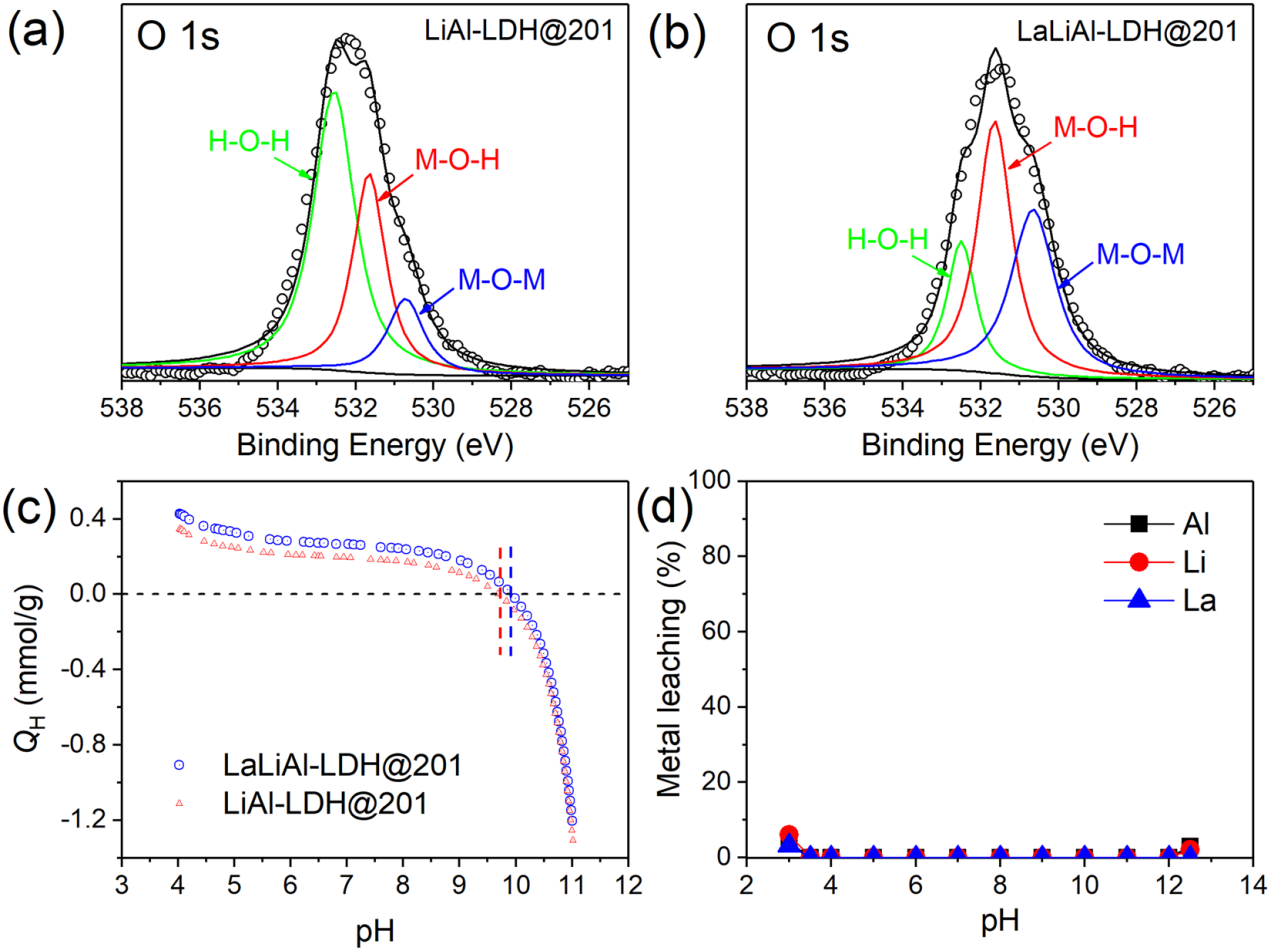
O 1 s XPS spectra of LiAl-LDH@201 (**a**) and LaLiAl-LDH@201 (**b**), pH-dependent surface charge of LiAl-LDH@201 and LaLiAl-LDH@201 (**c**), and pH-dependent metal leaching from LaLiAl-LDH@201 (**d**).

Comparing the XPS spectra of LiAl-LDH@201 and LaLiAl-LDH@201 (Fig. 3a,b), the O 1 s peak position shifted from 532.3 to 531.5 eV as a result of the La doping. The O 1 s spectra could be deconvoluted into three components at 530.7, 531.6, and 532.5 eV, respectively, corresponding to crystal O^2−^ in metal oxides (denoted as M-O-M), O atom in metal hydroxides or hydroxyl groups (denoted as M-O-H), and weakly adsorbed species (denoted as H-O-H), respectively^33–35^. Thus, after La doping, the fraction of M-O-H increased from 31.5% to 44.2%, which is believed to greatly favour the ligand exchange for fluoride uptake^36^. Accordingly, the slight increase in pH_pzc_ (from 9.6 to 9.8) and the more positive surface charge of LaLiAl-LDH@201 over LiAl-LDH@201 (Fig. 3c) also favour the attraction of F^−^. The leaching stability of LaLiAl-LDH@201 at varying pH was also explored (Fig. 3d). As observed, no leaching of La, Al, or Li was detected over broad pH range from 3.5 to 12. Such outstanding stability of LaLiAl-LDH@201 is expected to solve the regeneration issue of Al-based materials, since most of them cannot work at pH higher than 9.5 due to the inevitable Al leaching as well as the secondary pollution, as demonstrated by the desorption experiments below.

### Effect of pH

**Figure 4:**
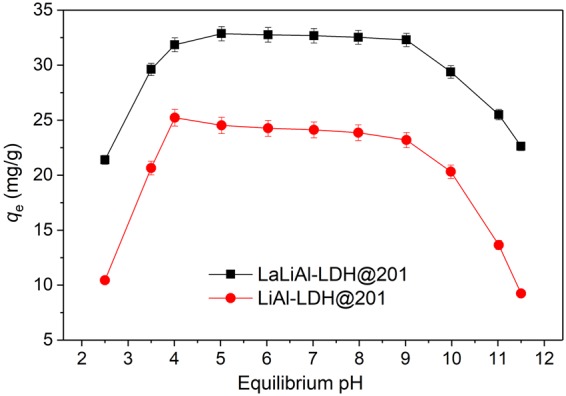
Effect of pH on fluoride adsorption by LiAl-LDH@201 and LaLiAl-LDH@201 at 298 K. [F^−^]_0_ = 20 mg/L, adsorbent dosage = 0.50 g/L, contact time = 24 h.

The effect of pH on the adsorption of fluoride by LiAl-LDH@201 and LaLiAl-LDH@201 are shown in Fig. 4. LaLiAl-LDH@201 showed satisfactory and stable fluoride uptake (~32 mg/g) over a wide range of pH from 4 to 9. At pH < 3, the fluoride uptake decreased sharply, possibly due to the formation of weakly ionised hydrofluoric acid (HF) and the potential dissolution of the LaLiAl-LDH. At pH > 10, the adsorption capacity also decreased rapidly because the adsorbent surface turned negatively charged (pH_pzc_ = 9.8), and the electrostatic repulsion of LaLiAl-LDH@201 and fluoride ions was unfavourable for its uptake. Similar pH-dependent fluoride adsorption performances were observed for LiAl-LDH@201 and LaLiAl-LDH@201. However, an evident enhancement of performance was observed due to the La doping, mainly owing to the specific interaction between F^−^ and the doped La(OH)_3_^23^, the interlayer expansion of LDH to allow more exchangeable Cl^−^ (Fig. S1), as well as the increased positive surface charge (Fig. 3c). Previous studies^17,23,37–39^ have suggested that many defluoridation adsorbents work only at weakly acidic pHs and suffer from deterioration in performance at weakly basic pHs (7.0–9.0). Thus, the broad applicable pH range for LaLiAl-LDH@201 (4–9) was of crucial significance for its potential application in view of the typical pH of groundwater (6.5–8.5).

### Effect of Competing Anions and Humic Acid

The influences of five types of ubiquitous anions (NO_3_^−^, Cl^−^, HCO_3_^−^, SO_4_^−^, and H_2_PO_4_^−^) on the adsorption of fluoride by LaLiAl-LDH@201 and LiAl-LDH@201 are shown in Fig. S2. For both adsorbents, the inhibitory influences of the examined anions followed the order of H_2_PO_4_^−^ > SO_4_^2−^ > HCO_3_^−^ > Cl^−^ > NO_3_^−^. Similar effects of the above competing anions on the adsorption of fluoride by LDH-based materials have been reported in our previous studies^16,29^ and elsewhere^14,40–42^. Although the effects of competing anions were similar for both LaLiAl-LDH@201 and LiAl-LDH@201, the La doping resulted in an evident increase in adsorptive capacity in the presence of the competing anions, indicating the enhancement of performance of the composite. The presence of humic acid exerted negligible influence on fluoride removal by LaLiAl-LDH@201 (Fig. S3), possibly due to the size exclusion effect of the mesoporous D201 host^43,44^.

### Adsorption Kinetics and Isotherm

The effect of contact time on fluoride uptake by LaLiAl-LDH@201 are presented in Fig. S4. As shown, a fast adsorption process occurred in the initial 50 min, and the adsorption equilibrium was achieved in ~400 min. The adsorption kinetics could be fitted with both pseudo-first order and pseudo-second order models with approximately identical coefficients of determination (*r*^2^ = 0.989 and 0.991, respectively). Also, as shown in the inset of Fig. S4, the experimental kinetic data could be described by the intra-particle diffusion model with two stages including diffusion and equilibrium, suggesting the intra-particle diffusion as the rate-determining step during the adsorption process.

The isotherm of fluoride adsorption onto LaLiAl-LDH@201 is shown in Fig. S5. Langmuir, Freundlich, and Sips models (detailed in Text S1 in Supplementary Information) were employed to simulate the experimental data, with the fitting parameters from the three models listed in Table S2. Comparatively, Freundlich model described the adsorption process better than Langmuir model, and Sips model implemented the best fit (*r*^2^ = 0.99). Preferential fitting by Sips and Freundlich models both with the heterogeneity parameter suggested that multi-site adsorption occurred during fluoride uptake by LaLiAl-LDH@201, where the immobilised LaLiAl-LDHs, La(OH)_3_, and the quaternary ammonium groups from the polymeric host D201 all served as active sites for fluoride sequestration. The adsorption capacity of LaLiAl-LDH@201 reached 75.7 mg/g based on Sips model calculation, which was larger than that of LiAl-LDH@201 (62.5 mg/g from Sips model) and other La-doped materials reported elsewhere (Table S3).

### Mechanism of Enhanced Adsorption

**Figure 5:**
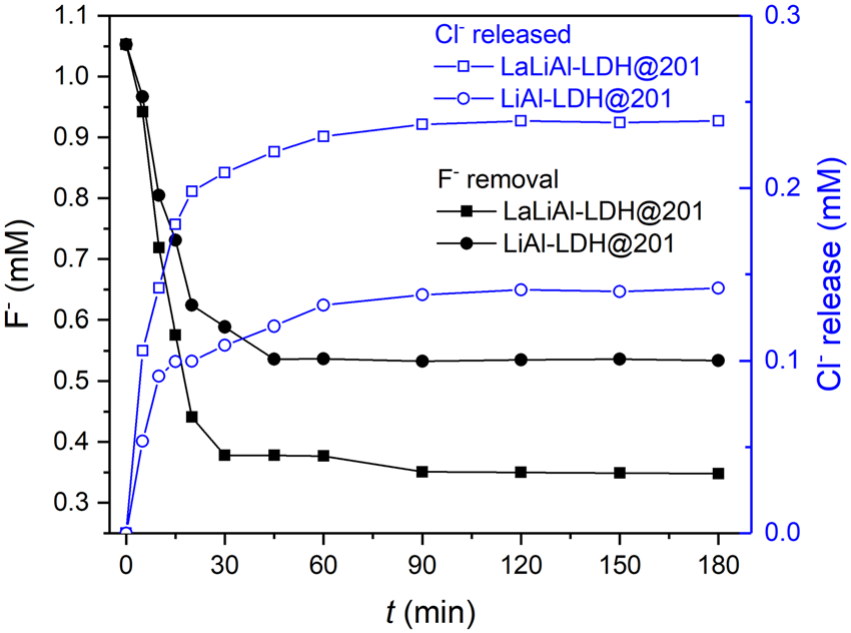
Release profile of chloride during fluoride uptake onto LaLiAl-LDH@201 and LiAl-LDH@201 at 298 K. [F^−^]_0_ = 20 mg/L, pH = 7.0 ± 0.2, adsorbent dosage = 0.50 g/L.

To verify the anion exchange of F^−^ for Cl^−^ in the immobilised LDHs during the sequestration of fluoride by LaLiAl-LDH@201, the release of Cl^−^ into solution as a function of time was monitored. As shown in Fig. 5, for the concentration of F^−^ was significantly inversely correlated with that of Cl^−^ (*r* = −0.969 for LaLiAl-LDH@201 and −0.956 for LiAl-LDH@201), indicating the intercalated Cl^−^ in the LDHs were exchanged by F^−^ for both adsorbents. More importantly, the amount of Cl^−^ released from LaLiAl-LDH@201 was also significantly higher than that from LiAl-LDH@201, consistent with the superior removal of F^−^ by La LiAl-LDH@201 over LiAl-LDH@201. This observation could support that the expansion of interlayer space due to La doping allowed more exchangeable Cl^−^ to be intercalated into the LDHs (Fig. S1), which promoted the fluoride adsorption capacity of the nanocomposite material.

**Figure 6:**
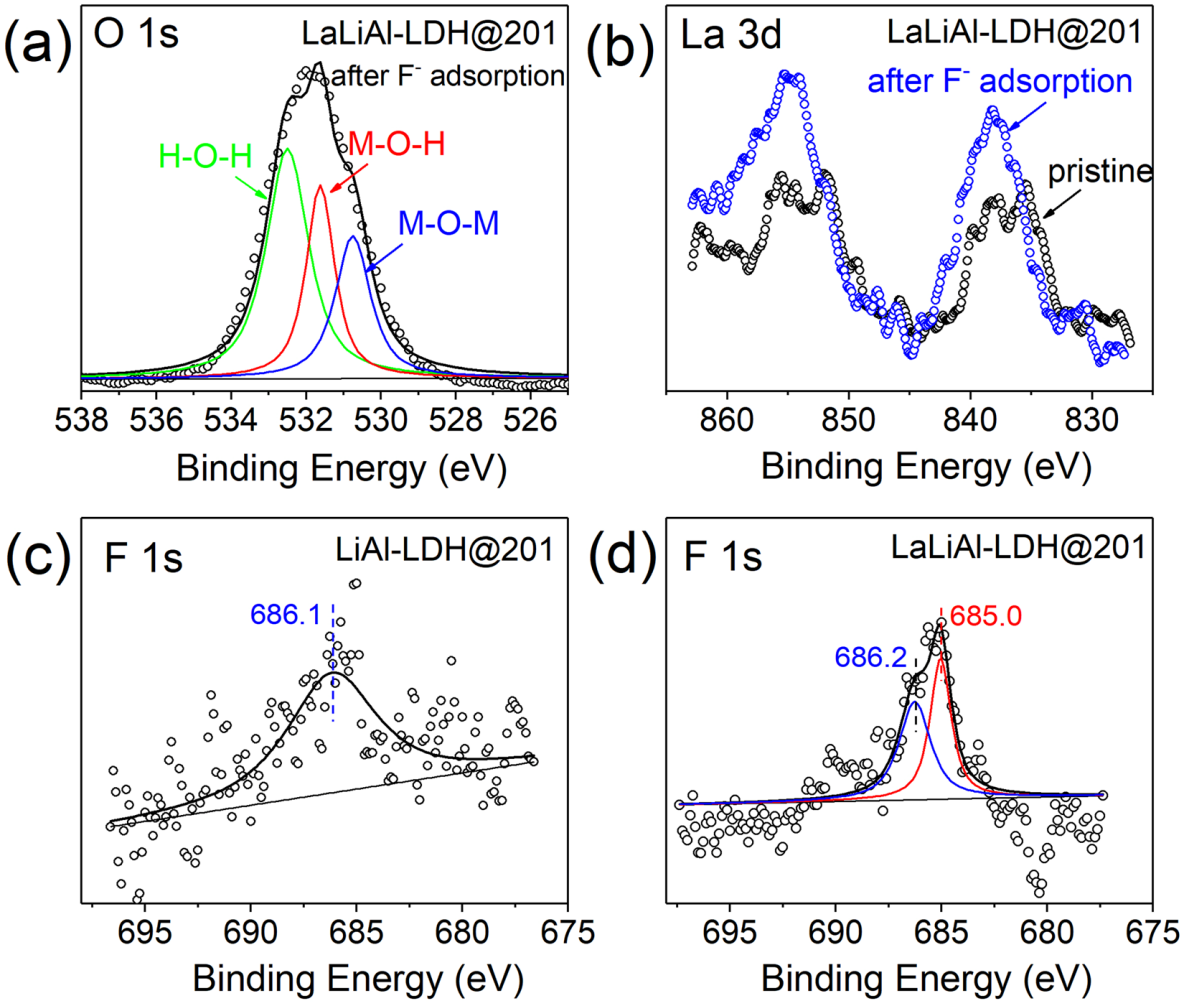
O 1 s, La 3d and F 1 s XPS spectra of fluoride-loaded LaLiAl-LDH@201 (**a**,**b**,**d**), and F 1 s spectrum of fluoride-loaded LiAl-LDH@201 (**c**).

XPS analysis were used to elucidate the adsorption interaction. As shown in Fig. 6a, the M-O-H fraction of LaLiAl-LDH@201 decreased from 44.3% to 37.7% after fluoride adsorption, indicating that ligand exchange occurred with the hydroxyl groups during fluoride uptake. Moreover, the La 3d_5/2_ and 3d_3/2_ peaks of LaLiAl-LDH@201 shifted to the higher-binding-energy side (Fig. 6b), suggesting the specific interaction between La and F. Meanwhile, the F 1 s spectra of LiAl-LDH@201 and LaLiAl-LDH@201 after fluoride uptake in the background of 500 mg/L SO_4_^2−^ are depicted in Fig. 6(c,d). The weak signal at 686.1 eV in the spectrum of LiAl-LDH@201 could be attributed to Al-F interaction, for most of the anion-exchange capability was compromised by SO_4_^2−^. For LaLiAl-LDH@201, the F 1 s signal was pronounced, and the main peak could be deconvoluted into two components at 686.2 eV and 685.0 eV, the latter one of which could be assigned to La-F interaction^25^. Thereby, the above observations on the La 3d and F 1 s XPS spectra indicated that the doped La played a significant role in F sequestration by LaLiAl-LDH@201through La-F bonding, which strengthened the affinity for fluoride.

**Figure 7:**
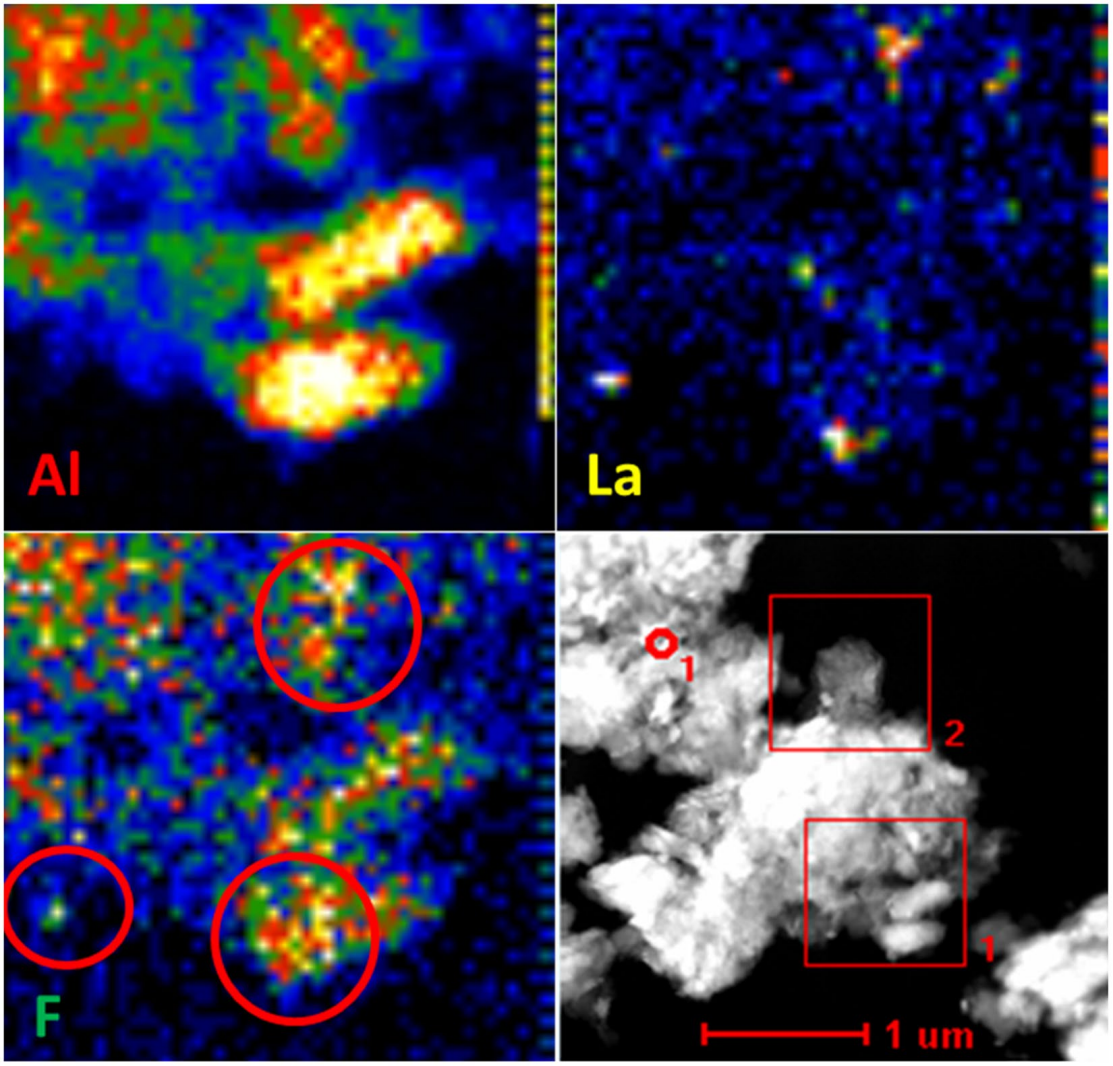
TEM-EDS mapping of Al, La, and F for fluoride-loaded LaLiAl-LDH@201.

TEM-EDS was employed to verify the above mechanism. As displayed in Fig. 7, the elemental distribution of the immobilised F on LaLiAl-LDH@201 was in good alignment with Al and La, and the specific La-F interaction could be observed in the highlighted area (red circled). The above results collectively indicated that the enhanced adsorption of fluoride by LaLiAl-LDH@201 was owing to both the specific affinity of the doped La for fluoride and the increased amount of exchangeable Cl^−^ in the embedded LDHs due to the La doping.

### Sustainable Fixed-Bed Defluoridation of Groundwater

**Figure 8:**
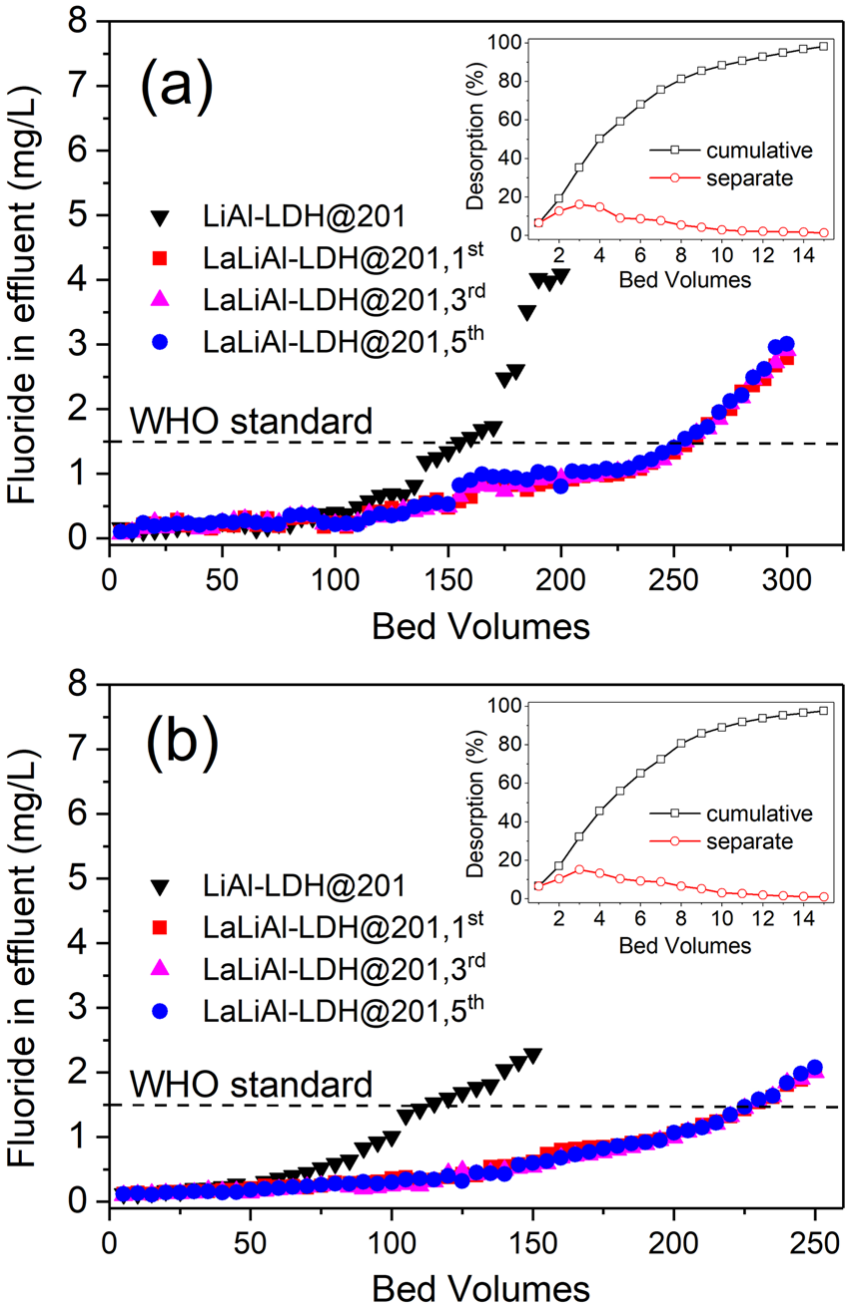
Breakthrough profiles of fluoride removal from groundwater samples from (**a**) Yunnan Province and (**b**) Shandong Province by and LiAl-LDH@201 and LaLiAl-LDH@201 (in five cyclic runs) at 298 K. Bed volume = 5 mL, empty bed contact time (EBCT) = 12 min. Insets show the respective desorption curves during the *in situ* regeneration processes.

The potential of the novel nanocomposite LaLiAl-LDH@201 for practical defluoridation was evaluated in fixed-bed column mode with two authentic fluoride-rich groundwater samples, whose detailed information is available in Table 1. Regarding the WHO standard (1.5 mg/L) as the breakthrough point, the effective treatment volume with LiAl-LDH@201 was 155 and 115 bed volumes (BV) for Sample 1 and Sample 2, respectively (Fig. 8). In contrast, the corresponding working capacities of LaLiAl-LDH@201 were increased to 255 and 230 BV, respectively. The larger treatable volume of LaLiAl-LDH@201 over LiAl-LDH@201 for the authentic fluoride-rich groundwater samples demonstrated the superiority of the novel composite induced by La doping. It is notable that with the influent fluoride concentration increased from 4.1 (Sample 1) to 10.0 mg/L (Sample 2), the fluoride uptake onto LiAl-LDH@201 increased from 2.8 mg/g to 4.5 mg/g, whereas that onto LaLiAl-LDH@201 increased from 4.5 mg/g to 10.7 mg/g. Through a convenient *in situ* regeneration process with 0.01 M NaOH and 1 M NaCl binary solution, the fluoride-loaded LaLiAl-LDH@201 could be effectively regenerated with desorption efficiency above 98% (inset of Fig. 8). Throughout five adsorption-regeneration cycles, no significant capacity loss of LaLiAl-LDH@201 was observed, and La leaching into the effluent was not detected. Furthermore, no significant difference was observed in the XRD spectra of LaLiAl-LDH@201 among the pristine material, the fluoride-loaded state, and the regenerated composite (Fig. 2), indicating that the crystal phase was stable during fluoride uptake and material regeneration. The high stability and constant working capacity suggested that LaLiAl-LDH@201 was competent for defluoridation of authentic groundwater in sustainable fixed-bed mode.

**Table 1:** Characteristics of the two fluoride-rich groundwater samples^a^.

Sample No.	Location	F^−^	Cl^−^	Br^−^	NO_3_^−^	SO_4_^2−^	HCO_3_^−^	Total P	Total Si	pH
1	Yunnan	4.1	74.6	ND^b^	16.7	67.2	41.2	0.11	0.21	8.15
2	Shandong	10.0	ND	1.5	0.08	169	213	0.05	13.5	7.95

^a^unit: mg/L except for pH.

^b^ND: not detected.

## Conclusions

A hybrid fluoride adsorbent was prepared by doping La into LiAl-LDH@201, denoted as LaLiAl-LDH@201. Material characterisation indicated La replaced a portion of Al in the octahedron located in the metal layer. The La incorporation expanded the interlayer space of the LDH, allowed more exchangeable Cl^−^ to be intercalated, while the stability of the sorbent remained unchanged. Compared to LiAl-LDH@201, LaLiAl-LDH@201 exhibited more satisfactory defluoridation performance. Such superior performance could be attributed to two adsorption mechanisms, which were increased capacity for anion exchange between Cl^−^ and F^−^, and the specific interaction between La and F. The fixed-bed working capacity of LaLiAl-LDH@201 for defluoridation of fluoride-rich groundwater was almost twice that of LiAl-LDH@201. LaLiAl-LDH@201 could be regenerated by NaOH + NaCl binary solution for sustainable defluoridation without notable capacity loss. This study signifies that a small proportion of La incorporation could evidently enhance F adsorption of LiAl-LDH supported by the polymeric anion-exchanger host, making La-doping a very cost-effective and promising way for improving F mitigation with LDH-based nanocomposites.

## Methods

### Materials

Humic acid sodium salt was purchased from Aladdin Reagent (Shanghai, China). All the other chemicals used in this study were of analytical grade from Sinopharm Chemical Reagent Co., Ltd. (Shanghai, China). Fluoride stock solution (20 mg F^−^/L) was prepared by dissolving desired amount of NaF in deionised water. The macroporous strongly basic anion exchanger D201 with polystyrene skeleton and quaternary ammonium groups was kindly provided by Jiangsu NJU Environmental Technology Co., Ltd. (Nanjing, China).

### Preparation of LaLiAl-LDH@201

The precursor nanocomposite LiAl-LDH@201, i.e., the nanocrystalline Li/Al LDHs impregnated D201, was fabricated via the reaction between Li^+^ and nanosized aluminium hydroxide inside the nanopores of D201 followed by hydrothermal treatment. Detailed information about its fabrication procedure is available in our previous study^16^. The novel La-doped nanocomposite LaLiAl-LDH@201 was prepared via a simple impregnation method. Briefly, the LaCl_3_ solution, which was prepared by dissolving 10.0 g LaCl_3_⋅7H_2_O in 60 mL binary solvent of deionised water and ethanol (5:1, v/v), was introduced into a flask containing 20 mL of LiAl-LDH@201 beads. Then, the suspension was subjected to continuous agitation at 343 K for 6 h. Finally, the resultant beads were filtrated, rinsed with deionised water, and dried at 323 K to obtain the nanocomposite LaLiAl-LDH@201. For comparison, powdered LaLiAl-LDH was prepared via co-precipitation method^29^.

### Characterisation

The crystalline structure of LaLiAl-LDH@201 was probed by X-ray diffraction (XRD, XTRA, Switzerland) with Cu Kα radiation (40 kV, 25 mA). A field emission transmission electron microscope (TEM, Tecnai F20, FEI, USA) with energy dispersive X-ray spectroscopy (EDS) and selected area electron diffraction (SAED) features was operated at 200 kV to characterise the morphology and microstructure of the adsorbents. The surface area and porous characteristics of the adsorbents were determined by N_2_ adsorption-desorption at 77 K on a surface and pore analyser (Nova 3000, Quantachrome, USA). The elemental contents of Li, Al, and La in the nanocomposite were quantified via HNO_3_-HClO_4_ digestion followed by aqueous determination with inductively coupled plasma optical emission spectrometry (ICP-OES, iCAP 7400, Thermo, USA). Elemental distribution on the cross-section of LaLiAl-LDH@201 was visualised by a scanning electron microscope (SEM, S-3400 II, Hitachi, Japan) coupled with EDS. X-ray photoelectron spectroscopy (XPS, PHI 5000 VersaProbe, Japan) was employed to characterise the states of the concerned elements with measured binding energies calibrated to the C 1 s peak at 284.8 eV.

### Batch Adsorption Experiments

The batch adsorption experiments were performed by dosing 0.50 g/L of adsorbent into 50 mL fluoride solution (typical concentration 20 mg/L) in polypropylene (PP) bottles, which were then shaken at 180 rpm in a thermostatic incubator at 298 K. The adsorption isotherms were obtained by varying the initial fluoride concentration from 10 to 50 mg/L. To investigate the effects of pH, coexisting anions (chloride, nitrate, sulphate, carbonate, and phosphate), and humic acid on fluoride uptake, the corresponding sodium salts were pre-introduced into the fluoride solution. The solution pH was adjusted with 0.10 M HCl or NaOH. The concentrations of Al, Li, and La leaching from the solid into the contact solution at various pHs were determined by ICP-OES. The kinetic experiments were carried out by placing 0.25 g of adsorbent into 500 mL fluoride solution initially at 20 mg/L. At preset time intervals, aliquots of 1 mL were withdrawn to analyse the concentrations of fluoride and chloride via ion-selective electrode (PF-2, Rex, China) and ion chromatography (ICS-1100, Dionex, USA), respectively.

### Fixed-bed Defluoridation of Groundwater and Adsorbent Regeneration

Fixed-bed column test was conducted by packing 5 mL of adsorbent into a glass column of 15 mm in diameter and 150 mm in length. Two fluoride-rich groundwater samples collected from Shandong and Yunnan provinces of China, were respectively used as the feeding water, which down flowed through the column at constant flow rate of 5 BV/h with a reciprocating pump. A binary solution of NaOH (0.01 M)-NaCl (1.0 M) was used to *in situ* regenerate the exhausted adsorbent at 2 BV/h. Five adsorption-regeneration cycles were conducted for each water sample to evaluate the sustainable defluoridation performance of LaLiAl-LDH@201 with LiAl-LDH@201 as comparison.

### Data availability

All data generated or analysed during this study are included in this published article (and its Supplementary Information files).

## Electronic supplementary material

Supplementary Information
