# Supplementary material for: Enhanced Defluoridation Using Novel Millisphere Nanocomposite of La-Doped Li-Al Layered Double Hydroxides Supported by Polymeric Anion Exchanger
Source: Sci Rep. 2018 Aug 6;8:11741. doi: 10.1038/s41598-018-29497-1 (PMC6079045; doi:10.1038/s41598-018-29497-1)
Supplement: Supplementary file 1 — Supplementary Information [file 41598_2018_29497_MOESM1_ESM.pdf]

Supplementary Information

Enhanced Defluoridation Using Novel Millisphere Nanocomposite of La-Doped Li-Al Layered Double Hydroxides Supported by Polymeric Anion Exchanger

Jianguo Cai[1,#], Yanyang Zhang[1,2,#], Yue Qian[1], Chao Shan[1,2,*], Bingcai Pan[1,2]

1. State Key Laboratory of Pollution Control and Resource Reuse, School of the Environment, Nanjing University, Nanjing 210023, China
2. Research Center for Environmental Nanotechnology (ReCENT), Nanjing University, Nanjing 210023, China

* Corresponding author.

E-mail: shanchao@nju.edu.cn.

[#] Both authors contributed equally to this work.

Text S1. Details of the isotherm models employed for fitting.

Langmuir, Freundlich, and Sips models are expressed as Eqs. (S1-S3). Sips model, also known as the Langmuir – Freundlich model, is based on the assumption that the distribution of binding affinities of the adsorption sites with the adsorbate can be described by a Gaussian-like distribution expression.

$$q_e = q_m \frac{bc_e}{1+bc_e} \tag{S1}$$

$$q_e = kc_e^{1/n} \tag{S2}$$

$$q_e = \frac{q_0 (b_0 c_e)^{\beta}}{1+(b_0 c_e)^{\beta}} \tag{S3}$$

where q_e (mg/g) and c_e (mg/L) are the fluoride concentration and the amount of fluoride adsorbed on unit mass of the adsorbent both at equilibrium, respectively. In Eq. (S1), q_m (mg/g) represents the adsorption capacity, while b (L/mg) is the Langmuir constant related to the adsorption energy. In Eq. (S2), n represents the heterogeneity of the sorbent and k is the Freundlich affinity constant. In Eq. (S3), q_0 (mg/g) and b_0 (L/mg) have similar meanings as q_m and b in Langmuir model, whereas β is an exponent introduced from Freundlich model to indicate the heterogeneity of the different adsorption sites. Smaller value of β suggests a wider affinity distribution and thus increasing the heterogeneity.

Table S1. General characteristics of LiAl-LDH@201 and LaLiAl-LDH@201.

Adsorbent	LiAl-LDH@201	LaLiAl-LDH@201
Skeleton of host	poly(styrene-divinylbenzene)	
Functional groups of host	$R\text{-}N^{+}(CH_3)_3$	
Pore volume (cm^3/g)	0.066	0.068
Average pore diameter (nm)	12.0	11.3
BET surface area (m^2/g)	24.8	26.7
Li/Al/La (molar ratio)	1/2.05/ --	1/1.89 /0.13

Table S2. Fitting parameters of the isotherms of fluoride adsorption on LaLiAl-LDH@201 at 298 K and pH 7.0±0.1.

Model	Parameter	Value
Langmuir	q_m (mg/g)	58.0
	b (L/mg)	0.28
	r^2	0.948
Freundlich	k (mg/g)	20.8
	n	3.85
	r^2	0.961
Sips	q_0 (mg/g)	75.7
	b_0 (L/mg)	0.30
	β	0.59
	r^2	0.990

Table S3. Comparison of fluoride adsorption capacity of LaLiAl-LDH@201 with other La-doped adsorbents.

Adsorbent	Capacity (mg/g)	Isotherm model	pH	*T* (K)	Reference
La-Al palygorskite	1.09	Langmuir	7.0	283	S1
La-Al-Scoria	1.25	Langmuir	7.2	283	S2
La-modified alumina	6.7	Langmuir	7.0	298	S3
La-loaded resin	24.5	Langmuir	6.0	303	S4
LaO_x-coated alumina	42.2	Langmuir	6.0	298	S5
LaLiAl-LDH	46.2	Langmuir	7.0	298	S6
Al-HA-La hydrogel	50.0	Langmuir	7.0	298	S7
LaLiAl-LDH@201	58.0<br>75.7	Langmuir<br>Sips	7.0	298	This study

References

S1 Lyu, Y., Su, X. S., Zhang, S. Y. & Zhang, Y. L. Preparation and characterization of La(III)-Al(III) co-loaded hydrothermal palygorskite adsorbent for fluoride removal from groundwater. *Water Air Soil Pollut.* **227**, https://doi.org/10.1007/s11270-016-3084-8 (2016).

S2 Zhang, S., Lu, Y., Lin, X., Su, X. & Zhang, Y. Removal of Fluoride from groundwater by adsorption onto La(III)- Al(III) loaded scoria adsorbent. *Appl. Surf. Sci.* **303**, 1-5, https://doi.org/10.1016/j.apsusc.2014.01.169 (2014).

S3 Cheng, J. M., Meng, X. G., Jing, C. Y. & Hao, J. M. La^{3+}-modified activated alumina for fluoride removal from water. *J. Hazard. Mater.* **278**, 343-349, https://doi.org/10.1016/j.jhazmat.2014.06.008 (2014).

S4 Luo, F. & Inoue, K. The removal of fluoride ion by using metal(III)-loaded amberlite resins. *Solvent Extr. Ion Exch.* **22**, 305-322, https://doi.org/10.1081/sei-120028007 (2004).

S5 Gu, H., Xu, Z., Li, L., Liu, F. & Zheng, S. Removal of fluoride from water using lanthanum oxide-coated alumina (in Chinese). *Acta Scientiae Circumstantiae* **29**, 589-593 (2009).

S6 Cai, J., Zhao, X., Zhang, Y., Zhang, Q. & Pan, B. Enhanced fluoride removal by La-doped Li/Al layered double hydroxides. *J. Colloid Interface Sci.* **509**, 353-359, https://doi.org/10.1016/j.jcis.2017.09.038 (2018).

S7 Liu, Y. *et al.* Adsorptive removal of fluoride from aqueous solutions using Al-humic acid-La aerogel composites. *Chem. Eng. J.* **306**, 174-185, https://doi.org/10.1016/j.cej.2016.07.036 (2016).

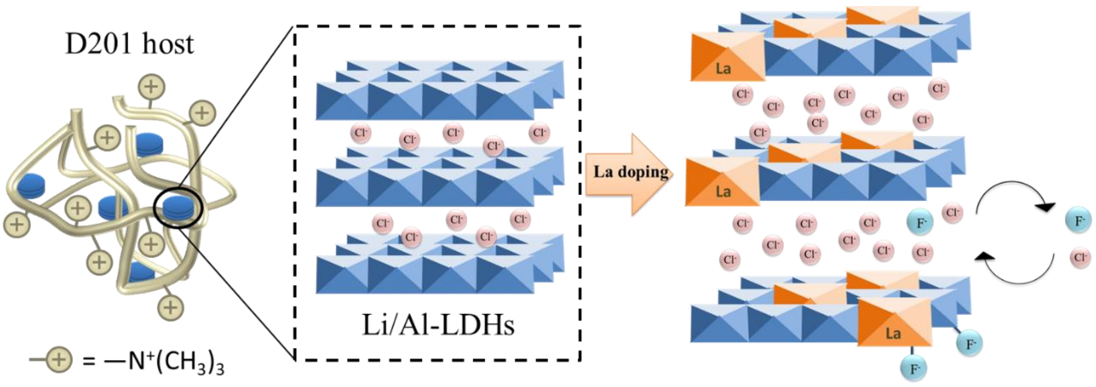

Figure S1. Schematic illustration of interlayer expansion due to La incorporation into LiAl-LDHs pre-immobilized inside the D201 polymeric host.

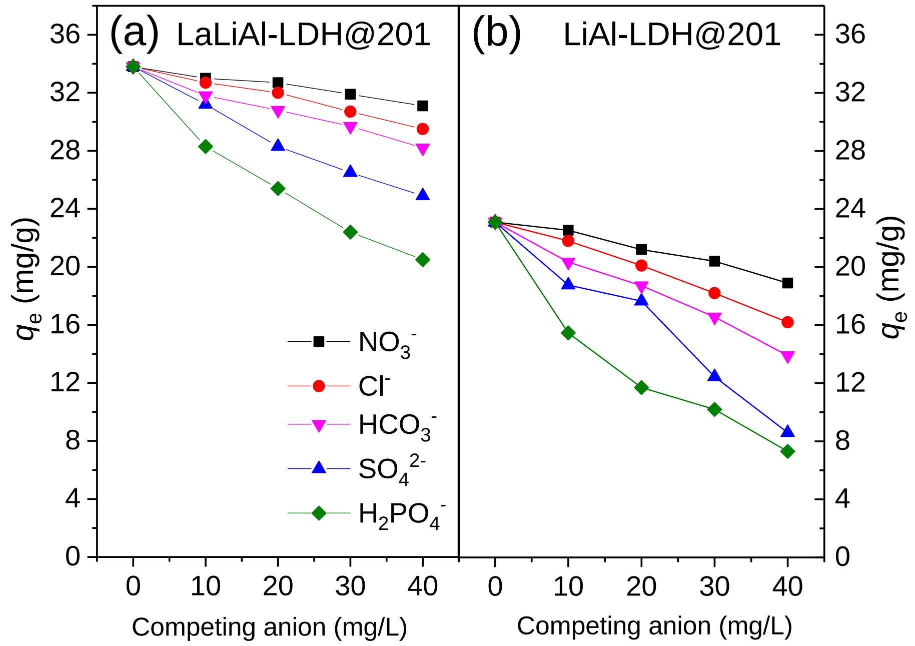

Figure S2. Effect of competing anions on fluoride adsorption by LaLiAl-LDH@201 and LiAl-LDH@201 at 298 K. $[F^-]_0$=20 mg/L, pH=7.0±0.2, adsorbent dosage=0.50 g/L, contact time=24 h.

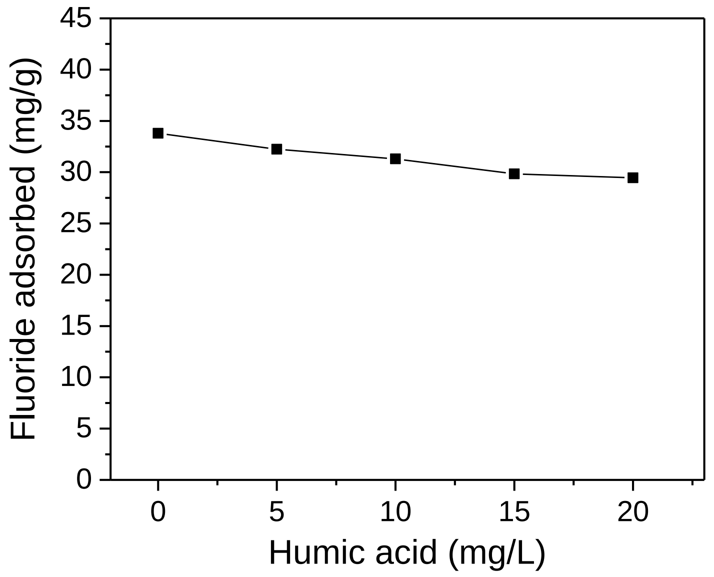

Figure S3. Effect of humic acid on fluoride uptake by LaLiAl-LDH@201. $[F^-]_0$=20 mg/L, pH=7.0±0.1, adsorbent dosage=0.50 g/L, contact time=24 h.

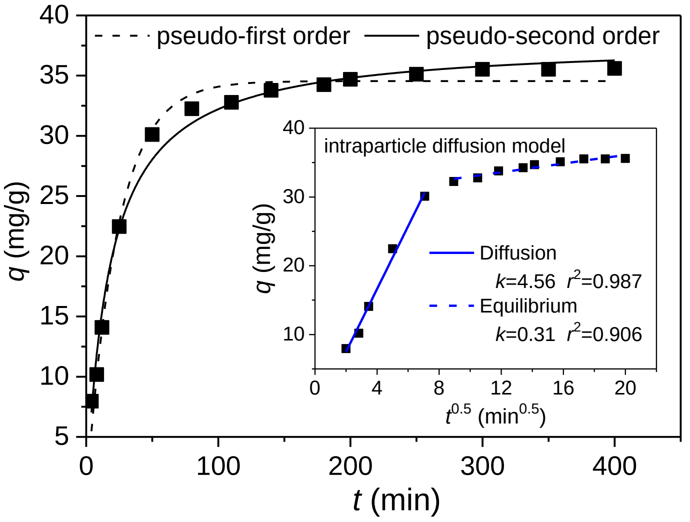

Figure S4. Adsorption kinetics of fluoride onto LaLiAl-LDH@201 at 298 K. $[F^-]_0$=20 mg/L, pH=7.0±0.1, adsorbent dosage=0.50 g/L.

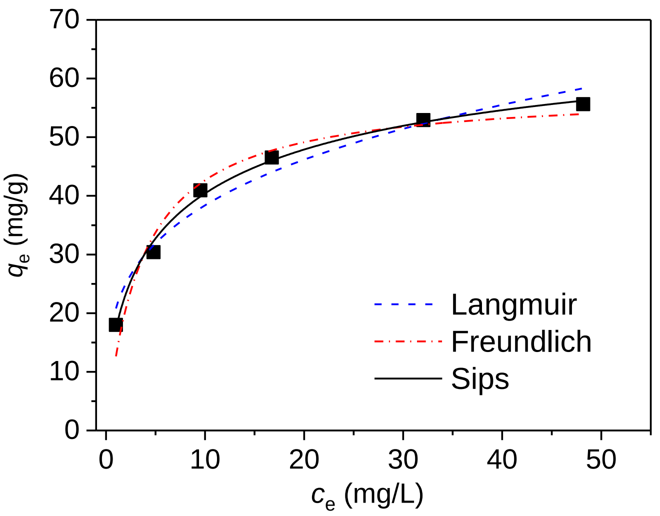

Figure S5. Adsorption isotherm of fluoride on LaLiAl-LDH@201 at 298 K. pH=7.0 ±0.1, adsorbent dosage=0.50 g/L, contact time=24 h.
